# Evaluation of an internet-based intervention for service members of the German armed forces with deployment-related posttraumatic stress symptoms

**DOI:** 10.1186/s12888-020-02595-z

**Published:** 2020-05-06

**Authors:** Helen Niemeyer, Christine Knaevelsrud, Sarah Schumacher, Sinha Engel, Annika Kuester, Sebastian Burchert, Beate Muschalla, Deborah Weiss, Jan Spies, Heinrich Rau, Gerd-Dieter Willmund

**Affiliations:** 1 Division of Clinical Psychological Intervention, Department of Education and Psychology, Freie Universität Berlin, Freie Universität Berlin, Schwendenerstr. 27, 14195 Berlin, Germany; 2 Department of Clinical Psychology, Psychotherapy and Diagnostics, Institute of Psychology, Technische Universität Braunschweig, Braunschweig, Germany; 3 German Armed Forces, Military Hospital Berlin, Department for Military Mental Health, Berlin, Germany

**Keywords:** Posttraumatic stress disorder, Service members, Military, Internet-based intervention, Efficacy

## Abstract

**Background:**

The present study was designed to evaluate the efficacy of a therapist-guided internet-based cognitive-behavioral therapy (iCBT) intervention for service members of the German Armed Forces with posttraumatic stress disorder (PTSD). The iCBT was adapted from Interapy, a trauma-focused evidence-based treatment based on prolonged exposure and cognitive restructuring. It lasted for 5 weeks and included 10 writing assignments (twice a week). The program included a reminder function if assignments were overdue, but no multimedia elements. Therapeutic written feedback was provided asynchronously within one working day.

**Methods:**

Male active and former military service members were recruited from the German Armed Forces. Diagnoses were assessed with the Clinician-Administered PTSD Scale for DSM-5 (CAPS-5) and the Mini-International Neuropsychiatric Interview. Psychopathology was assessed at pre-treatment, post-treatment, and 3-month follow-up. Severity of PTSD was the primary outcome and anxiety was the secondary outcome. Participants were randomly allocated to a treatment group that received iCBT immediately or to a waitlist group that received iCBT after 6 weeks. Due to the overall small sample size (*n* = 37), the two groups were collapsed for the statistical analyses. Change during the intervention period was investigated using latent-change score models.

**Results:**

Improvements in the CAPS-5 were small and not statistically significant. For anxiety, small significant improvements were observed from pre- to follow-up assessment. The dropout rate was 32.3%.

**Conclusions:**

The low treatment utilization and the high dropout rate are in line with previous findings on treatment of service members. The interpretation of the current null results for the efficacy of iCBT is limited due to the small sample size, however for military samples effect estimates were also smaller in other recent studies. Our results demonstrate the need to identify factors influencing treatment engagement and efficacy in veterans.

**Trial registration:**

Australian Clinical Trials Registry ACTRN12616000956404.

## Background

### Posttraumatic stress disorder in military personnel

Posttraumatic stress disorder (PTSD) is a common disorder, with a 12-month prevalence rate of 3.7% in the US general population [1] and 2.3% in the German general population, with the latter being in line with PTSD prevalence rates in other European countries [2]. Military personnel have an elevated risk of experiencing or being exposed to traumatic stressors such as threat to one’s own person or colleagues [3] and witnessing suffering and death [4–7]. Point prevalence rates vary between 2 and 17% for US veterans and 3 to 6% for British veterans after deployment, according to a systematic review [8]. A 12-month prevalence rate of 2.9% was found for combat-experienced service members of the German Armed Forces (Bundeswehr [9]). These varying prevalence rates might be explained by factors such as frequency and duration of deployments, but also by cross-national differences in military structures or in the openness to disclose sensitive information about PTSD symptoms [10–13]. Within the German Armed Forces, the risk of developing PTSD is increased in combat-experienced military personnel as compared to never-deployed military personnel [14], and only one in two service members with PTSD are diagnosed or treated [9].

Deployment-related PTSD is often accompanied by depression, anxiety, and substance misuse [4, 15], functional impairments [16], relationship difficulties [8], and poor quality of life [17]. If left untreated, it often follows a chronic course [6]. Moreover, military personnel with subthreshold PTSD are also at risk of worsening psychological distress [18, 19].

### Psychotherapeutic treatment for service members with PTSD

Given the significant impairment associated with subclinical and clinical levels of PTSD, access to efficacious interventions is important. International evidence-based guidelines recommend trauma-focused cognitive behavioral therapy (TF-CBT) and eye movement desensitization and reprocessing (EMDR) for the treatment of PTSD [20, 21]. For face-to-face TF-CBT compared to control groups a standardised mean difference of 1.62 was found in a comprehensive meta-analysis [22]. However, also according to meta-analytic evidence, military samples benefit less from such interventions than civilians, although TF-CBT still shows stronger effects than other psychotherapeutic approaches, with symptom reductions from pre- to post-test of *g* = 1.06 [23–28].

Moreover, between 60 and 75% of veterans with PTSD do not seek treatment [29–33], and among those who do, the number of attended sessions is usually low. Studies indicate that only 2–10% of veterans suffering from PTSD complete the treatment as intended [30, 34].

Reasons for not seeking treatment, dropping out, or not optimally benefitting include stigma, confidentiality concerns and perceived treatment inefficacy [10, 35–37]. Logistical and access barriers and concerns about potential negative effects of treatment-seeking on one’s military career also influence treatment-seeking behavior [37, 38]. Face-to-face treatments require appointments in an outpatient clinic or a hospital for which patients often need to take time off from work. Studies demonstrated that fear of judgment and exclusion by comrades and the leadership as well as fear of negative consequences for the career due to psychotherapeutic treatment and psychiatric diagnoses are widespread among military personnel, and that confidentiality concerns play an important role in not seeking treatment or dropping out of it [10, 37, 39–42].

Taken together, low rates of treatment utilization, high dropout rates and lower efficacy of treatments in veterans highlight the need to optimize treatment for PTSD in military service members [26]. Service members might benefit from more flexible treatment options that protect their privacy. The low threshold and visual anonymity of internet-based treatments have the potential to reach specific populations that otherwise might not seek treatment, e. g. individuals with fear of judgment or stigmatization. Distance delivery approaches such as internet-based interventions provide access to evidence-based treatments such as TF-CBT [26], minimize treatment barriers and increase client confidentiality [43, 44], especially when participants do not have to meet a mental health care professional in person before starting an internet-based intervention. Participants can access the treatment in their own time and while staying in their personal environment, e.g. while being at home.

In internet-based CBT (iCBT), evidence-based treatment protocols are delivered online, generally based on asynchronous written communication [45]. The content is usually not altered, deviating from face-to-face CBT only in the method of delivery [46]. Most notably, Lange and colleagues [47] developed a pioneering iCBT for trauma victims by combining a manual-based cognitive-behavioral writing therapy with the Internet (Interapy), but also other internet-based programs have taken a trauma-focused CBT approach. ICBT is easily accessible and privacy sensitive. It also aims at reducing healthcare expenditures [46]. Moreover, iCBT has been found to be acceptable and compatible with the establishment of a good therapeutic relationship in civilians [48, 49]. Therefore, iCBT can fill an important gap [32].

### Efficacy of iCBT in military personnel

US veterans appear to be receptive to the use of mental health technologies [50, 51]. A preliminary study of a CBT-based online workshop (afterdeployment.org) supplemented with weekly telephone calls reported an effect size of *d* = 1.04 [52]. A study evaluating a mobile app intervention (PTSD coach) was perceived as helpful [53].

A meta-analysis revealed that iCBT for PTSD was more efficacious than waitlist (*d* = 0.95), although it was not found to be superior to active comparison interventions [54]. However, only one study included in the meta-analysis has been based on a military sample (DESTRESS [55]). DESTRESS comprised six writing assignments focusing on cognitive restructuring and trauma exposure. Compared to an active control group, a small effect size of *d* = 0.41 at post-treatment emerged. The effect disappeared at the 3-month follow-up (*d =* 0.10) but was large at the 6-month follow-up (*d* = 0.95). The dropout rate lay at 30% [55].

In a more recent trial, a modified version of DESTRESS without writing assessments but with homework and telephone support was compared to treatment as usual. Of the 491 primary care patients approached for study participation, about half (49%) refused [56]. Thirty-five percent of the participants completed the treatment. The effect size at post-treatment (*d* = 0.23) and at 12-week follow-up (*d* = 0.47) was small, and disappeared after 18 weeks (*d* = 0.08 [56]).

Another recent study compared an iCBT program for veterans with PTSD, named “Vets Prevail”, with treatment as usual [57]. Vets Prevail did not include trauma exposure and no writing assignments, but psychoeducation, media elements, individualized storylines, serious gaming principles and real-time chats with other veterans. It resulted in a small effect size at post-test, and the dropout rate lay at 20% (*d* = 0.42 [57]). To the best of our knowledge, these are all Randomized Controlled Trials (RCTs) on the efficacy of iCBT in military personnel that have been published to date.

To sum up, all studies compared iCBT to active control conditions, and the writing-based version of DESTRESS as well as Vets Prevail yielded at least small effects. All studies were conducted in the US. As military structures and prevalence rates of PTSD differ cross-nationally, it is worthwhile to investigate the efficacy of iCBT for service members in military structures other than the US.

### Treatment of PTSD in the German armed forces

The German Armed Forces provide treatment in military hospitals, and civilian psychotherapists are also involved in delivering treatment to military personnel [58]. However, treatment utilization rates are lower than the prevalence and incidence rates for German military personnel with PTSD, with less than 50% of traumatized service members seeking treatment within 12 months after deployment [9].

A number of treatments for PTSD offered by the German Armed Forces have demonstrated good efficacy. Non-trauma-focused inpatient group CBT showed medium efficacy from pre- to post-treatment (*d* = 0.68) and high efficacy to follow-up (*d* = 0.95 [59]). For inpatient EMDR, a medium pre-post effect size of *d* = 0.77 was found [60]. However, there is a need for comprehensive and low-threshold treatment within the German Armed Forces.

### Objectives

The current study was a RCT designed to investigate the feasibility, acceptability and efficacy of iCBT in service members of the German Armed Forces. In view of the findings demonstrating the efficacy of iCBT, we adapted a trauma-focused, therapist-guided iCBT [61, 62] that was based on the treatment protocol of Interapy [47] and Integrative Testimonial Therapy (ITT [63]). Service members experiencing mild to severe posttraumatic distress, but also with chronic courses, were suitable for inclusion. The iCBT was compared to a waitlist (WL) condition, and we expected a moderate reduction of posttraumatic stress symptoms. Anxiety was assessed as the secondary psychopathological outcome. We hypothesized that treatment effects would be sustained during the 3-month follow-up period. In accordance with previous findings, it was expected that participation rates would be lower than in civilian samples.

## Methods

### Sample and measures

Participants were deemed eligible for the current study if they met all of the following criteria: 1) male members of the German Armed Forces with clinical PTSD according to Diagnostic and Statistical Manual of Mental Disorders-5 (DSM-5) [64], or with subclinical symptoms, that is symptoms on one or more subscales of the CAPS without the overall number of symptoms for the clinical diagnosis. In addition, the person had to report suffering from these symptoms and report to be in need of treatment. 2) Minimum age of 18 years (no restrictions on maximum age); 3) active or out-of-duty service members; 4) fluency in reading and writing in the German language; 5) ability to use computers without assistance and 6) regular access to the internet for the duration of the iCBT.

Exclusion criteria were acute psychosis, acute manic episode, current substance abuse or dependence, current suicidal ideation, neurological disorder, acute somatic disease, concurrent psychotherapeutic treatment, or unstable psychotropic medication.

An eligibility telephone screening comprised the assessment of the PTSD A criterion (traumatic event) and the PTSD Checklist for DSM-5 (PCL-5) [65], followed by the Mini International Neuropsychiatric Interview (M.I.N.I) sections for alcohol and substance abuse, psychotic symptoms, depressive and manic episode [66]. Suicidality was assessed with the respective Beck Depressions-Inventar-II (BDI-II) item (item 9 [67]). Finally, we asked whether the participant was in current psychotherapeutic or pharmacological treatment.

At each diagnostic face- to-face assessment, symptom severity (primary outcome) and the presence of a diagnosis of PTSD were assessed by applying the German translation of the Clinician-Administered PTSD Scale for DSM-5, which has very good psychometric properties (CAPS-5 [68]). The CAPS-5 is an interview-based assessment of all PTSD domains. Each item is rated by a clinician on a 5-point scale ranging from 0 = *not present* to 4 *= extreme*, representing the severity of PTSD symptoms during the last month. The CAPS yields an overall score (range: 0 to 80) as well as subscale scores reflecting symptom severity in the PTSD core domains (Criterion B: Re-experiencing symptoms, max. Twenty points; Criterion C: Avoidance symptoms, max. Eight points; Criterion D: Negative alterations in cognitions and mood, max. Twenty-eight points; Criterion E: Alterations in arousal and reactivity, max. twenty-four points). Comorbid diagnoses were assessed with the Mini-International Neuropsychiatric Interview German Version 5.0, a structured clinician-administered diagnostic interview according to DSM-IV and ICD-10 (M.I.N.I [69]).

Traumatic events were assessed at the first assessment with the Life Events Checklist for DSM-5 (LEC-5 [70, 71];), which assesses exposure to 16 selected types of potentially traumatic events (e.g., severe accident, severe physical injury) and provides the additional option to report any other potentially traumatic event. The List of the Mental Health Advisory Team (LMHAT [72]) was also used at the first assessment to assess 33 military- and deployment-related traumatic events.

Sociodemographic information was also collected (e.g., age, relationship status, education, length of duty in the armed forces) at the first assessment, and medical records and previous psychotherapeutic, pharmacological, and medical treatments were documented.

The secondary outcome anxiety was measured at each assessment using the 7-item Generalized Anxiety Disorder scale (GAD-7, [73]). It assesses anxiety symptoms during the past 2 weeks and has good psychometric properties [74]. Moreover, participants completed a number of self-report questionnaires (e.g. about posttraumatic cognitions, posttraumatic growth, moral injury) at each assessment.

The risk of suicide (measured by the BDI-II item number 9 [67]) was repeatedly assessed, that is before the first writing assignment as well as during the course of treatment, i.e. after the third (biography), seventh (exposure) and tenth (cognitive restructuring) writing session. After the treatment, patients were also asked about potential adverse events during the course of treatment. Adverse effects were assessed with the Negative Effects Questionnaire [75].

### Procedure

The sample was recruited via advertisements in military journals, on websites and in online chat rooms for service members. Printed flyers and posters were distributed in health service centers and military hospitals of the German Armed Forces. Service members were also recruited by coordinating with unit commanders, who distributed flyers in post-deployment seminars. The study was presented at mental health conferences of the German Armed Forces to military psychologists and psychiatrists.

Service members could contact the study team via email or telephone. An appointment was scheduled for a pre-consent eligibility screening in a telephone call by a licensed therapist (BM, JS). The telephone screening took about 45 min. If a participant was found to be eligible in the screening, he was invited to the German Armed Forces Hospital Berlin for a full diagnostic assessment.

Participants were randomly assigned to the immediate treatment group (IT) or the waitlist control group (WL) before the first diagnostic assessment. Randomization was based on a computer-generated randomization list in excel. Written consent was obtained. Participation was voluntary and strictly confidential. Patients received no financial reward for their participation. All eligible active service members were released from their routine duty for the assessments, without knowledge of their seniors about participation in a treatment study.

The diagnostic assessment was conducted by clinical psychologists (AK, SB, BM) or graduate Master’s-level psychology students (HK, CKE, DW) who were especially trained in administering the CAPS-5 (CAPS-5 [68]) and the M.I.N.I. (M.I.N.I [69]). The assessors were not blinded.

After the diagnostic interviews had been conducted the trauma event checklists were provided, which were administered on a computer screen. The pre-treatment assessment also included an introduction to the website’s structure, and participants were provided with a login code and set a personal password. Post-treatment self-report questionnaires were assessed partly online and partly during the face-to-face post-assessment in the hospital 1 week after the end of the intervention.

Assessments in the German Armed Forces Hospital Berlin were completed three (IT) to four (WL) times (pre-treatment, post-treatment, 3-month follow-up). The WL control group attended an additional pre-wait time assessment, followed 6 weeks later by the pre-treatment assessment. This waiting interval was chosen because of the treatment duration, which was 5 weeks, plus 1 week to consider also the days between the pre-treatment assessment and the start of the treatment as well as between the end of the treatment and the post-treatment assessment. The assessments took 1 day and required overnight stays of participants with a longer travelling time to the German Armed Forces Hospital in Berlin. Therefore, the assessments were scheduled for either 1 or 2 days, depending on the arrival times of the participants on the first day. For a description of the comprehensive study design see Supplement 1.

**Fig. 1:**
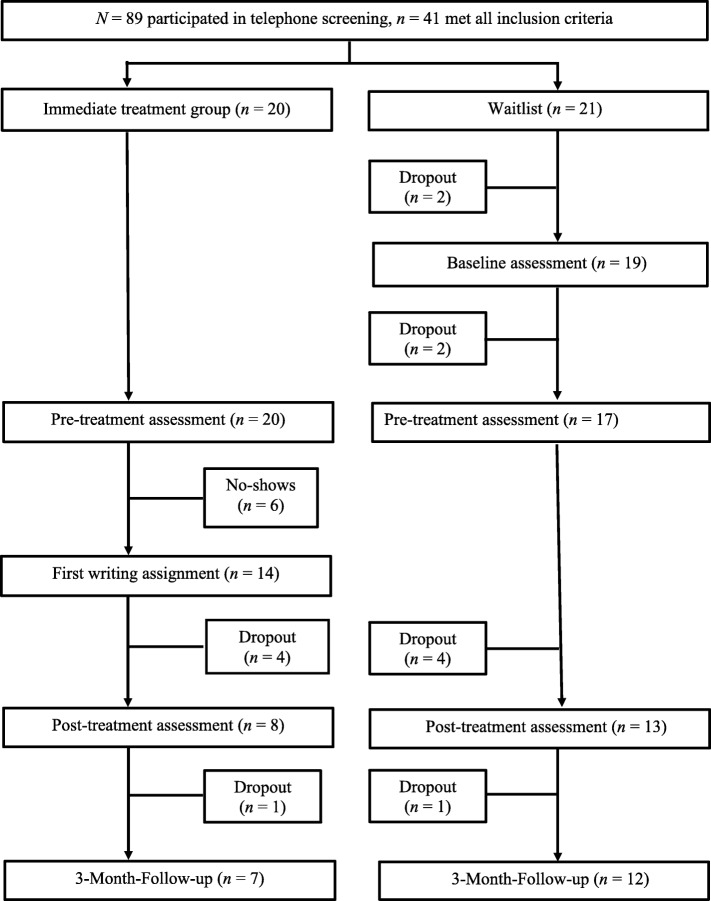
Participants flow chart. Descriptive data on the patient flow through the study, that is the number of participants and drop-outs. Provides all details of the participant flow from the telephone screening to the follow-up assessment, which comprises also the detailed numbers of drop-outs per group and time-point (that is, according to the intervals between the assessments)

All participants received treatment within 1 week after the pre-treatment assessment. The post-treatment assessment was completed within 1 week after the final session of the iCBT. The WL group received the same treatment after the waiting period. Details of participant flow are shown in Fig. 1.

Of 89 service members who were screened, 41 met all inclusion criteria and were randomized to either the IT (*n* = 20, 48.8%) or WL (*n* = 21, 51.2%) group. Two of the WL participants did not attend the pre-wait time assessment, and another two withdrew during the waiting period. Altogether, 37 participants started the intervention.

Six (16.2%) participants were classified as no-shows. Ten (32.3%) participants who began the treatment dropped out (*n* = 21 completers).

Data were collected between July 2016 and July 2018. The study was approved by the Freie Universität Berlin Institutional Review Board [reference number: 85/2014].

### Intervention

The iCBT lasted for 5 weeks, and participants were instructed to write twice a week (10 essays in total). Each writing assignment required approximately 45 min of writing time. The therapists conducting the treatment were female postdoctoral-level psychologists licensed in cognitive-behavioral psychotherapy (HN, SSch) who had received special training in therapeutic writing via the internet. They provided written feedback after one working day and were available on demand in case of questions about the interventions or for technical support via phone calls.

The iCBT consisted of three treatment phases: 1) biographical reconstruction, 2) exposure, and 3) cognitive restructuring. In the first treatment phase of the biographical reconstruction, in three writing assignments, the patient described his childhood, youth and adulthood up to the time shortly before the most debilitating traumatic event. Previous studies demonstrated the relevance of reflecting on positive life experiences, but also on negative experiences which patients have already successfully overcome in life [63].

The second treatment phase comprised repeated exposure to the most debilitating traumatic event in four writing assignments. Participants received psychoeducation about the mechanisms of exposure and were instructed to describe the traumatic event in the first person and present tense. The therapists helped the patients to focus on the most painful aspects and the emotions, thoughts and sensory perceptions that they experienced during the traumatic event.

The third phase comprised three sessions of cognitive restructuring. In order to develop a new perspective on the traumatic event, patients wrote supportive letters to their former self at the time shortly after they had experienced the traumatic event. They were instructed to reflect on feelings of guilt and shame, to challenge dysfunctional automatic thinking and behavior patterns and to correct unrealistic assumptions. Patients were also encouraged to consider potentially positive consequences of the traumatic event and lessons learned from it, and to reflect on how they plan to cope with it in the future. The participants were instructed not to concentrate on style, grammar or spelling in their writing assignments, and were assured of the confidentiality of their writing.

Written feedback by the therapist was provided after one working day for all writing tasks except for session 2 (biographical reconstruction) and session 5 (exposure). Feedback for these sessions was combined with the feedback for the following session and thus provided after sessions 3 and 6, respectively. All feedback was based on standardized templates from the treatment manual, which were tailored within the boundaries of the protocol to patients’ specific needs. Important aspects of this feedback were recognition and reinforcement of the patients’ work, positive feedback and motivation, as well as help and directions if the biographical reconstruction, the exposure or the cognitive restructuring had not been performed as intended. To address the needs of German service members, we tailored the treatment to their specific situation and provided the treatment in the German language.

Reminders were sent if assignments were overdue. Contact on demand was possible to clarify questions about the intervention or in the case of technical problems. Any patient who did not respond to the reminder or who reported suicidal ideation during the iCBT was contacted by one of the study coordinators (BM, JS). The study’s safety protocol included a timeline for contacting the patient and instructions on how to assess the risk of suicidality and to take appropriate action if necessary. The intervention was provided via an encrypted communication platform.

### Statistical analyses

An a priori power analysis (power = 95%, alpha *=* 5%, two-tailed) indicated that at least 100 participants are needed to estimate a moderate between-group difference of *d* = 0.7, based on results of previous meta-analyses. Due to recruitment issues, only *n* = 37 persons started the intervention (WL = 17, IT = 20), and a relatively large proportion of non-completion (43%) occurred. Therefore, we decided to collapse the two groups to investigate symptom change during the intervention period. Even though this results in uncontrolled effect-estimates we deem this to give a better idea of how individuals change than a between group-comparison based on two very small group with large rates of attrition.

We compared no-shows (individuals who did not start the treatment) with treatment-starters (individuals who completed at least the first writing assignment) and dropouts (individuals who started but did not complete the treatment) with completers using Fisher’s exact tests for categorical variables and Welch-tests for continuous variables.

Change in PTSD symptoms and in anxiety over the course of treatment was estimated using latent change score (LC) models [76–78] using Mplus 8.0, which is comparable to computing a *t*-test for repeated measures [79]. We favored this approach due to the ease of using multiple imputation to deal with a large proportion of missing values.

An intention-to-treat (ITT) analysis was performed, that is, all participants randomized and available immediately prior to the start of the intervention were included.

Given the large proportion of missing values, we used different approaches for dealing with missing data. First, we used last observation carried forward (LOCF). Since no between-group comparisons are made, this approach can be considered as conservative, under the assumption that individuals who drop out do not deteriorate. Second, we used multiple imputation as implemented in the *R* package MICE on the level of subscale scores (50 datasets, 50 iterations, [80]. Two different imputation methods were used: *predictive mean matching* (MI-PMM) and *norm* (MI-norm), since the best practice to impute large proportions of missing values in small data sets is still a matter of debate. Finally, a completer analysis was performed including only those individuals who completed all 10 treatment modules. The different approaches used to deal with missing data help to gain an impression of how estimated effects change under various assumptions (sensitivity analysis). We report unstandardized mean changes, which are better comparable across measurement occasions because they are unaffected by differences in the variability of change. In addition, we computed standardized effect size by dividing the mean change score by its standard deviation (d). We also assessed clinically meaningful changes from pre- to post-test as well as from pre-test to follow-up. Changes in the CAPS-5 score of > / = 10 were considered as clinically meaningful and percentages of improvement, non-improvement, and worsening were calculated [81].

All analyses were two-sided and *p* < .050 indicated statistical significance. Figures were created in *R* using *ggplot* [82].

## Results

The mean age of the combined sample was 37.7 years (*SD* = 9.8; range: 19–70). 80.6% of the participants were in full-time employment while participating in the treatment and had served in the German Armed Forces for an average of 17 years (*SD* = 9.9; range: 1–52). On average, they had participated in 2.8 foreign missions (*SD* = 3.1; 0–15), mostly in Afghanistan (73.5%). Table 1 gives a summary of the sample characteristics.

**Table 1:** Sociodemographic Characteristics

			Comparison of groups			
Variables		Total (*n* = 37)	Waiting List (*n* = 17)	Treatment Group (*n* = 20)	Test statistics *t* (*df*)	*p*
Sociodemographic characteristics						
Age	*M* (*SD*)	37.7 (8.8)	37.8 (12.8)	37.7 (6.86)	0.01 (21.79)	.989
Marital status^a^						.786
Single	*n* (*%*)	4 (11.1)	1 (6.2)	3 (15)		
Relationship	*n* (*%*)	5 (13.9)	2 (12.5)	3 (15)		
Married	*n* (*%*)	19 (52.8)	10 (62.5)	9 (45)		
Education^a^						.680
Secondary school qualification	*n* (*%*)	6 (16.7)	3 (18.8)	3 (15.0)		
Secondary school certificate	*n* (*%*)	24 (66.7)	11 (68.8)	13 (65)		
High school diploma	*n* (*%*)	6 (16.7)	2 (12.4)	4 (20)		
Employment status^a^						.894
Fulltime	*n* (*%*)	29 (80.6)	13 (81.2)	16 (80)		
Part-time	*n* (*%*)	1 (2.8)	0 (0)	1 (5)		
Training/Apprenticeship	*n* (*%*)	1 (2.8)	0 (0)	1 (5)		
Unemployed	*n* (*%*)	5 (13.9)	3 (18.8)	2 (10)		
Joined the military	Year (*SD*)	2001 (9.9)	2000 (12.5)	2001 (7.6)	−0.06 (21.87)	.950
Work status^a^						.593
Voluntary service	*n* (*%*)	2 (6.1)	2 (12.5)	0 (0)		
Temporary	*n* (*%*)	20 (60.6)	10 (62.5)	10 (58.8)		
Professional	*n* (*%*)	8 (24.2)	3 (18.8)	5 (29.4)		
Military unit^a^						.145
Army	*n* (*%*)	18 (51.4)	10 (62.5)	8 (42.1)		
Air Force	*n* (*%*)	5 (14.3)	0 (0.0)	5 (26.3)		
Navy	*n* (*%*)	1 (2.9)	1 (6.2)	0 (0)		
Medical service	*n* (*%*)	3 (8.6)	1 (6.2)	2 (10.5)		
Joint support service	*n* (*%*)	8 (22.9)	4 (25.0)	4 (21.1)		
Military ranks						
Enlisted ranks	*n* (*%*)	10 (28.6)	5 (31.2)	5 (26.3)		
Non-commissioned	*n* (*%*)	20 (57.1)	10 (62.5)	10 (52.6)		
Commissioned officer	*n* (*%*)	5 (14.3)	1 (6.2)	4 (21.1)		
Number of deployments	*M* (*SD*)	2.78 (3.12)	1.75 (1.18)	3.60 (3.9)	−2.01 (23.24)	.056
Country^a^						.634
Afghanistan	*n* (*%*)	25 (73.5)	9 (60.0)	16 (83.2)		
Bosnia	*n* (*%*)	2 (5.9)	1 (6.7)	1 (5.3)		
Kosovo	*n* (*%*)	5 (14.7)	3 (20.0)	2 (10.5)		
Mali	*n* (*%*)	1 (2.9)	1 (6.7)	0 (0)		
Somalia	*n* (*%*)	1 (2.9)	1 (6.7)	0 (0)		

*Note*. ^++^Fisher′s exact test was used to test the significance of independence in categorical variable. The category “secondary school qualification” (Realschule) also includes “subject-restricted higher education entrance qualification” (Fachhochschulreife). *df* degrees of freedom, *M* mean, *N* sample size, *PTSD* posttraumatic stress disorder, *SD* standard deviation

Overall, 94.4% of the participants reported combat or war zone exposure, followed by witnessing severe human suffering (88.6%), as assessed with the LEC-5. The most debilitating traumatic event had occurred on average 9.4 years ago (*SD* = 5.8; range 2.0–25.0). Furthermore, according to the LMHAT scale, service members were frequently exposed to several traumatic events (mean: 33.4, *SD* = 17.2, range 8.0–74.0; see also Supplement 2).

The mean CAPS-5 score was 33.5 (*SD* = 14.9). Twenty-two participants (59.5%) had a PTSD diagnosis. Comorbid mood and anxiety disorders were common: 13 patients (31.5%) suffered from comorbid major depression and *n* = 7 (18.9%) reported dysthymia. Two patients (5.41%) had experienced a lifetime manic episode and *n* = 1 (2.7%) a lifetime hypomanic episode, but reported no current symptoms. Nine patients (33.3%) suffered from panic disorder, *n* = 19 (51.4%) from agoraphobia, *n* = 7 (18.9%) from social phobia and *n* = 5 patients (13.5%) from generalized anxiety disorder. There were no differences in comorbid diagnoses between the former IT and WL group. The majority of the sample had previously received psychotherapeutic or pharmacological treatment (58.3%; see Table 2).

**Table 2:** PTSD psychopathology and treatment

		Comparison of groups				
Variables	Total (*n* = 37)	Waiting List (*n* = 17)		Treatment Group (*n* = 20)	Test statistics *t* (*df*)	p
Clinical PTSD (CAPS)						1.000
No	*n* (*%*)	15 (40.5)	7 (41.2)	8 (40)		
Yes	*n* (*%*)	22 (59.5)	10 (58.8)	12 (60)		
CAPS sum score	*M* (*SD*)	33.54 (14.88)	33.6 (15.3)	33.3 (1)	0.06 (33.76)	.954
Previous treatment						0.320
Yes	*n* (*%*)	21 (58.3)	11 (68.8)	10 (50)		
No	*n* (*%*)	15 (41.7)	5 (31.2)	10 (50)		
Current pharmacological treatment						1.000
Yes	*n* (*%*)	9 (25)	4 (25)	5 (25)		
No	*n* (*%*)	27 (75)	12 (75)	15 (75)		

*Note*. Previous treatment was psychotherapeutic or psychiatric treatment. *df* degrees of freedom, *M* mean, *N* sample size, *PTSD* posttraumatic stress disorder, *SD* standard deviation

### No-shows and dropout

While no-shows did not differ from participants who began treatment on any clinical or sociodemographic characteristic, all no-shows were randomized to the IT condition. There were no significant differences between dropouts and completers (see Supplement 3). Reasons reported for dropout comprised difficulties with internet connection (*n* = 1), lack of motivation for treatment (*n* = 1), preferring face-to-face over online settings (*n* = 4), feeling no improvement (*n* = 2), and hospital admission (*n* = 1). One participant did not report a reason for dropout.

Adverse effects during the iCBT where assessed in the completer sample and 9.5% reported severe resistance against the writing assignments, whereas another 23.8% experienced intense negative feelings while they were writing.

### Pre-treatment to post-treatment changes

**Fig. 2:**
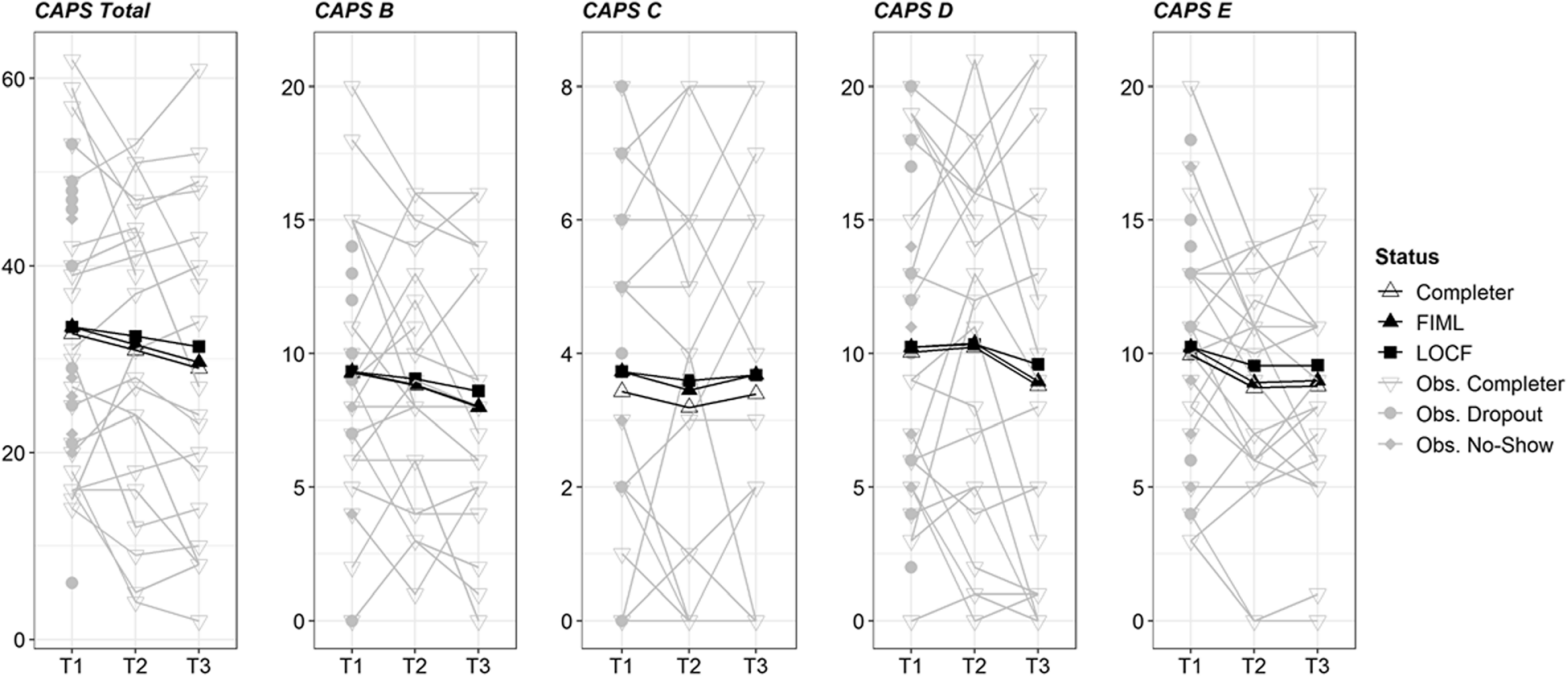
Individual trajectories. Individual trajectories of the change in PTSD symptoms over the course of the treatment. Shows the individual trajectories and the estimated mean changes in the CAPS-5 total and subscale-scores. The results for completers, the results from the last observation carried forward (LOCF) approach, and the results from the full information maximum likelihood (FIML) are displayed

Within-group effect size estimates revealed no significant changes for any of the measured outcome variables from pre- to post-assessment (see Table 3). The estimated average changes as measured with the CAPS-5 total score ranged between − 1.0 (LOCF) and − 2.35 (MI-PMM), depending on the method that was applied to handle missing data. Overall, the CAPS-5 total score was missing for 43% (*n* = 16) at post-assessment and for 49% (*n* = 18) at follow-up-assessment. Figure 2 shows the individual trajectories and the estimated mean changes in the CAPS-5 total and subscale-scores. The percentage of clinical meaningful change from pre- to post-test as well as from pre-test to follow-up is described in Table 4. The majority of participants did not change (about two thirds), and more participants improved than deteriorated.

**Table 3:** Changes in Outcome Measures Across the Intervention

Outcome	Analysis	*n*	Pre	Pre-to-Post				Pre-to-Follow-Up			
				D	[95% CI]	*p*	*d*	D	[95% CI]	*p*	*d*
CAPS Total	Completer	21	32.71	−1.76	[−5.71, 2.19]	.388	−0.19	−3.71	[−7.48, 0.92]	.086	−0.38
	LOCF	37	33.54	−1.00	[−3.35, 1.27]	.394	−0.14	−2.11	[−4.54, 0.38]	.096	−0.27
	MI PMM	37	33.54	−2.35	[−6.55, 1.84]	.272	−0.21	−5.42	[− 10.51, − 0.33]	.037	− 0.42
	MI NORM	37	33.54	−1.68	[− 5.11, 1.75]	.337	−0.19	− 3.28	[− 7.01, 0.45]	.085	− 0.36
CAPS - B	Completer	21	9.29	−0.48	[−1.95, 1.00]	.525	−0.14	−1.29	[−2.63, 0.00]	.053	−0.44
	LOCF	37	9.32	−0.27	[−1.11, 0.60]	.532	−0.10	−0.73	[−1.54, 0.03]	.066	−0.30
	MI PMM	37	9.32	−0.54	[−2.17, 1.10]	.520	−0.13	−0.93	[−2.48, 0.62]	.239	−0.26
	MI NORM	37	9.32	−0.41	[−1.92, 1.10]	.596	−0.11	−1.21	[−2.54, 0.13]	.076	−0.39
CAPS - C	Completer	21	3.43	−0.24	[−0.86, 0.52]	.513	−0.14	− 0.03	[− 0.78, 0.70]	.930	−0.02
	LOCF	37	3.73	−0.14	[−0.54, 0.27]	.515	−0.11	− 0.05	[− 0.49, 0.35]	.798	−0.04
	MI PMM	37	3.73	−0.42	[−1.32, 0.48]	.357	−0.20	−0.36	[− 1.52, 0.79]	.538	− 0.13
	MI NORM	37	3.73	−0.28	[−1.01, 0.45]	.448	−0.16	−0.04	[− 0.80, 0.72]	.924	− 0.02
CAPS - D	Completer	21	10.05	0.19	[−1.43, 2.14]	.833	0.05	−1.25	[−3.50, 1.21]	.299	−0.24
	LOCF	37	10.24	0.11	[−0.84, 1.19]	.833	0.04	−0.65	[−1.84, 0.65]	.311	−0.17
	MI PMM	37	10.24	0.08	[−1.89, 2.05]	.937	0.08	−2.27	[−5.23, 0.69]	.132	0.32
	MI NORM	37	10.24	0.20	[−1.62, 2.01]	.834	0.05	−0.91	[−3.41, 1.59]	.475	−0.18
CAPS - E	Completer	21	9.95	−1.24	[−2.57, 0.14]	.070	−0.39	−1.17	[−2.43, 0.12]	.076	−0.40
	LOCF	37	10.24	−0.70	[−1.49, 0.05]	.081	−0.28	−0.68	[−1.46, 0.05]	.078	−0.29
	MI PMM	37	10.24	−1.47	[−3.03, 0.09]	.065	−0.37	−1.85	[− 3.59, − 0.11]	.037	−0.44
	MI NORM	37	10.24	−1.18	[−2.63, 0.26]	.109	−0.35	−1.13	[− 2.54, 0.28]	.116	−0.36
GAD-7	Completer	21	11.76	−1.53	[−3.81, 0.29]	.147	−0.35	−2.79	[−5.66, − 0.75]	.022	− 0.62
	LOCF	30	12.30	−1.23	[−2.67, −0.03]	.065	−0.33	−2.20	[−3.87, − 0.83]	.005	− 0.51
	MI PMM	37	11.87	−1.66	[−4.15, 0.83]	.191	−0.27	−3.04	[−5.59, − 0.48]	.020	− 0.58
	MI NORM	37	11.96	−1.34	[−3.62, 0.93]	.247	−0.28	−2.22	[− 4.35, − 0.09]	.041	− 0.54

*Note*. *Completer* analysis of working with all 10 treatment modules, *LOCF* Missing data dealt using last observation carried forward, *MI NORM* ITT with multiple imputed data using norm, *MI PMM* ITT with multiple imputed data using predictive mean matching, *D* average change in metric of the questionnaire

**Table 4:** Rates of individuals showing clinical meaningful change (Change in CAPS overall score > = 10)

Outcome	Analysis	*% improved*	Post *% unchanged*	*% deteriorated*	*% improved*	Follow-up *% unchanged*	*% deteriorated*
CAPS Total	Completer	28.6	61.9	9.5	26.3	63.2	10.5
	LOCF	16.2	78.4	5.4	16.2	78.4	5.4
	MI PMM	31.1	55.5	13.4	36.7	51.2	12.1
	MI NORM	22.9	67.8	9.2	24.9	66.0	9.1

^a^ Please note that the rates for LOCF are equal for both measurement occasions. This is because LOCF assumes no change for cases with missing data per definition

### Pre-treatment to 3-month follow-up changes

Regarding the CAPS-5 scores, the different methods mainly indicated that the changes between pre-treatment and 3-month follow-up were not significant. Depending of the approach to deal with missing data, the estimated average improvements as measured with the CAPS-5 total score ranged between 2.11 (LOCF) and 5.42 (MI-PMM) points. All methods indicated a significant symptom reduction regarding the secondary outcome GAD-7 ranging from − 2.20 (LOCF) to − 3.04 (MI-PMM) points. Table 3 summarizes all estimates.

## Discussion

The present study aimed to investigate the acceptability and efficacy of iCBT in a German military sample with subclinical or clinical levels of PTSD. Only 37 service members completed the pre-treatment assessment. Six individuals did not begin the treatment and 10 individuals dropped out during the course of treatment. Investigating the change occurring during the intervention period resulted in small and non-significant changes as assessed with the CAPS-5. This is also true for individuals who completed the intervention, and changes from pre-treatment to 3-month follow-up were also non-significant. Small, significant improvements from pre- to follow-up assessment emerged for anxiety.

Overall, only *n* = 89 of individuals could be screened for eligibility. The most frequently reported reasons for dropout were preferring face-to-face over online settings and lack of improvement. When assessing adverse effects, we found that up to 25% of the completers reported intense negative feelings during the iCBT. It should be emphasized that trauma-focused, exposure-based interventions are often experienced as aversive, and thorough psychoeducation as well as cognitive restructuring of dysfunctional thoughts about trauma exposure are necessary to convince patients about the treatment [83]. In particular, service members might need an individual discussion of potential fears about facing memories of traumatic events. In the current iCBT, such in-depth psychoeducation was probably not possible to the required extent. Notably, many participants in the current study had difficulties writing the trauma-exposure assignments in the present tense, and in focusing on their emotions in detail, which might hint at trauma avoidance. Potential reasons discussed in the literature influencing treatment-seeking behavior and dropout in military personnel in face-to-face interventions include trauma avoidance and comorbidity [84, 85, 86, 87–90]. The first meta-analysis on predictors of treatment efficacy found that the number of trauma-focused therapy sessions predicted effectiveness and that both high and low pre-treatment PTSD severity levels predicted lower treatment gains [25].

In the current study, some participants also had difficulties connecting to the internet and accessing the web page, which in some cases required repeated personal instruction (telephone consultations) to resolve. The patients mainly used the contact on demand option for technical support. Questions about the assignments were usually resolved in the written feedback. Reading and writing skills, interest in writing, as well as computer skills may have hampered the motivation to begin and complete the iCBT. Additionally, the rather effortful assessment days required participants to travel to the German Armed Forces Hospital, in some cases with overnight stays, and the home sampling of psychophysiological markers (see Supplement 2) demanded time and preparation. Moreover, despite the fact that confidentiality was protected the assessments in the military hospital might have elicited subjective concerns in some participants. Therefore, the advantages of iCBT were probably less accessible for the current sample. Future studies on the acceptability and efficacy of iCBT might benefit from phone-based instead of face-to-face diagnostic assessments.

An evaluation of how iCBT can be successfully promoted in military systems could be helpful, as policies concerning confidentiality remain an ongoing issue. An additional reason might include disability compensation incentives. Furthermore, many participants in the current study had received psychotherapeutic treatment before and presented rather chronic symptoms, and might have developed low expectations about the efficacy of treatment in general.

Our findings are in line with the low utilization and high dropout rates reported for military personnel in psychotherapeutic treatment in general and partly consistent with the previous studies on iCBT in military personnel with PTSD. Treatment efficacy was comparably higher for the written-based DESTRESS version and for Vets Prevail than in our trial. However, due to a number of differences between the interventions and the study designs, the results are not directly comparable. The intervention in the current study was text-intensive, while Vets Prevail, in contrast, included sophisticated media elements. Vets Prevail was investigated in non-active-duty veterans with mild to moderate symptoms, including also females [57]. The majority of the male participants in the current study had been confronted with several army-associated traumatic events, many were particularly burdened, and a considerable number were still in active duty. Furthermore, all previous studies on iCBT were conducted in the US, which has different military structures and higher PTSD prevalence rates.

Broadening the perspective beyond military personnel, a recent meta-analysis investigating the efficacy of iCBT for PTSD in both non-military and military samples [46] found lower effect estimates compared to previous meta-analyses. This demonstrates the need for future studies to identify patient and treatment characteristics that modify treatment success. In order to improve iCBT, high quality clinical trials that systematically disentangle the role of different program components and patient characteristics for the acceptance and efficacy of iCBT are necessary. With respect to the identification of patient characteristics that modify treatment acceptance and success, sociodemographic and psychopathological characteristics, such as clinical status, symptom severity and traumatic events, should be investigated systematically. Moreover, examining the impact of specific variations of program components such as the duration of treatment, therapeutic support, and technical components such as multimedia components or reminders, as well as additional modules such as stress management, for example, can help to identify who benefits most from which iCBT concept (see also [54]).

Another promising way to improve iCBT are blended approaches, such as combining face-to-face treatment with iCBT or iCBT with mobile applications. Blended approaches may be particularly indicated for patients with deficits in emotion regulation or stress management. The practice of new skills can be prompted in everyday life, for example to help to cope with negative emotions, and the higher treatment intensity and support in the application of therapeutic strategies might enhance the acceptability and efficacy. However, more research in general and especially in military samples is necessary.

Moreover, utilization is likely determined by a complex interaction between patient, treatment, and system factors, and embedding new approaches such as iCBT within an already given care setting is also crucial. An evaluation how new modes of delivery such as iCBT can successfully be promoted especially in military systems could be helpful.

### Limitations

We employed a randomized controlled design, but collapsed the groups and reported uncontrolled estimates even though we were aware that it is important to follow the study plan [88]. The interpretability of the results is still compromised by the small sample size and the missing data. Further analysis of potentially relevant predictors of treatment efficacy or dropout was not possible due to the small sample size.

### Future studies

Our results highlight priorities for future studies. Considering that iCBT is cost-efficient and easily accessible, possibilities to promote the advantages as well as leverage strategies, such as motivational interviewing, should be investigated. If iCBT is to be helpful, it must be acceptable. Future research should focus on identifying participant and intervention features that are relevant for treatment efficacy.

## Conclusion

Military members present unique challenges in the treatment of PTSD. This study represents a call to action to validate interventions to improve treatment engagement and retention. Progress in the field is unlikely to occur without a better understanding of patient preferences and factors influencing treatment engagement and retention. Fostering engagement and willingness to remain in psychotherapeutic treatment is essential to ensure the provision of evidence-based treatment to military personnel. Future research in this regard is eagerly encouraged.

## Supplementary information

**Additional file 1.** Comprehensive study design including psychophysiological and experimental assessments.

**Additional file 2.** Supplement 2. Traumatic events.

**Additional file 3.** Supplement 3. Sociodemographic Characteristics for No-shows vs. ITT sample and Dropouts vs. Completers.

## Data Availability

The data that support the findings of this study are available upon reasonable request from the German Federal Ministry of Defence (BMVg FüSK III 5).

## Abbreviations

ACTRN: Australian Clinical Trials Registry
AK: Annika Kuester
BDI-II: Beck depressions-inventar II
BM: Beate Muschalla
BMVg FüSK: German Federal Ministry of Defence
CAPS-5: Clinician-Administered PTSD Scale for DSM-5
CKE: Christina Kersjes
CK: Christine Knaevelsrud
DSM-5: Diagnostic and statistical manual of mental disorders-5
DW: Deborah Weiss
EMDR: Eye Movement desensitization and reprocessing
GAD: Generalized anxiety disorder scale
GDW: Gerd-Dieter Willmund
HK: Hannah Klusmann
HN: Helen Niemeyer
HR: Heinrich Rau
iCBT: Internet-based cognitive-behavioral therapy
IT: Immediate treatment group
ITT: Intention-to-treat
JS: Jan Spies
LC: Latent change score
LEC-5: Life events checklist for DSM-5
LMHAT: List of the Mental Health Advisory Team
LOCF: Last observation carried forward
M.I.N.I: Mini International Neuropsychiatric Interview
MI-norm: Imputation method - norm
MI-PMM: Imputation method - predictive mean matching
PCL-5: PTSD Checklist for DSM-5
PTSD: Posttraumatic stress disorder
RCT: Randomized controlled trial
SB: Sebastian Burchert
SE: Sinha Engel
SSCH: Sarah Schumacher
TF-CBT: Trauma-focused cognitive behavioral therapy
WL: Waitlist control group
