# Supplementary material for: Evaluation of an internet-based intervention for service members of the German armed forces with deployment-related posttraumatic stress symptoms
Source: BMC Psychiatry. 2020 May 6;20:205. doi: 10.1186/s12888-020-02595-z (PMC7204035; doi:10.1186/s12888-020-02595-z)
Supplement: Supplementary file 1 — Additional file 1. Comprehensive study design including psychophysiological and experimental assessments. [file 12888_2020_2595_MOESM1_ESM.docx]

**Comprehensive study design including psychophysiological and experimental assessments**

The investigation of treatment efficacy was embedded in a comprehensive study design to investigate mechanisms of change and dysregulations in various psychophysiological systems.

As secondary outcomes, biological markers reflecting the regulation of the hypothalamic-pituitary-adrenal (HPA) axis, markers of the autonomic nervous and immune system, dehydroepiandrosterone, oxytocin and vasopressin were assessed. Additionally, at each diagnostic face-to-face appointment, eye-tracking assessments were conducted to measure attentional bias, and heart rate variability was assessed.

In an additional cross-sectional study arm, the PTSD patients were compared at baseline to never-traumatized (NE) as well as trauma-exposed (TE) healthy controls with respect to potential dysregulations in the psychophysiological systems, attentional bias and heart rate. Inclusion criteria for the healthy participants were male adult German-speaking service members of the German Armed Forces who had either experienced a potentially traumatic event (TE) or had never experienced a potentially traumatic event (NE), but did not fulfill the criteria for PTSD or any other mental disorder. Exclusion criteria were neurological disorders and acute physical illness. The results of these secondary outcomes will be reported elsewhere.
