# Supplementary material for: Evaluation of an internet-based intervention for service members of the German armed forces with deployment-related posttraumatic stress symptoms
Source: BMC Psychiatry. 2020 May 6;20:205. doi: 10.1186/s12888-020-02595-z (PMC7204035; doi:10.1186/s12888-020-02595-z)
Supplement: Supplementary file 2 — Additional file 2. Supplement 2. Traumatic events. [file 12888_2020_2595_MOESM2_ESM.docx]

| Supplement 2. Traumatic events | | | | | | | |  |  |  |
| --- | --- | --- | --- | --- | --- | --- | --- | --- | --- | --- |
| Variables | *Sample n* _total_ | *N event†* | % | Variables | *n_total_* | *n* | % | |  |  |
| LEC5 | | | | Seeing dead or seriously injured comrades 34 21 | | | 61.76 | |  |  |
| Natural disaster | 36 | 20 | 55.56 | Knowing someone seriously injured/killed | 34 | 29 | 85.29 | |  |  |
| Fire or explosion | 36 | 30 | 83.33 | Participating in demining operations | 34 | 15 | 44.12 | |  |  |
| Transportation accident | 36 | 31 | 86.11 | Improvised explosive device/booby trap exploded nearby | 33 | 20 | 60.61 | |  |  |
| Serious accident | 36 | 21 | 58.33 | Working in mined areas | 34 | 22 | 64.71 | |  |  |
| Exposure to toxic substance | 36 | 9 | 25 | Having hostile reactions from civilians | 34 | 27 | 79.41 | |  |  |
| Physical assault | 36 | 21 | 58.33 | Disarming civilians | 34 | 14 | 41.18 | |  |  |
| Assault with a weapon | 36 | 30 | 83.33 | Shooting/directing fire at enemy | 34 | 20 | 58.82 | |  |  |
| Sexual assault | 36 | 4 | 11.11 | Calling in fire on the enemy | 34 | 8 | 23.53 | |  |  |
| Other unwanted sexual experience | 36 | 2 | 5.56 | Engaging in hand-to-hand combat | 34 | 6 | 17.65 | |  |  |
| Combat/exposure to a war-zone | 36 | 34 | 94.44 | Clearing/searching homes/buildings | 34 | 13 | 38.24 | |  |  |
| Captivity | 36 | 3 | 8.33 | Clearing/searching caves/bunkers | 34 | 6 | 17.65 | |  |  |
| Life-threatening illness/injury | 36 | 21 | 58.33 | Witness of mistreatment of uninvolved persons | 34 | 12 | 35.29 | |  |  |
| Severe human suffering | 35 | 31 | 88.57 | Being wounded/injured | 34 | 7 | 20.59 | |  |  |
| Sudden violent death | 36 | 27 | 75 | Seeing injured women or children, but unable to help | 34 | 18 | 52.94 | |  |  |
| Sudden accidental death | 36 | 20 | 55.56 | Receiving incoming artillery/rocket/mortar fire | 34 | 21 | 61.76 | |  |  |
| Serious injury/harm/death caused to someone | 36 | 8 | 22.22 | Being responsible for enemy combatant’s death | 32 | 5 | 15.63 | |  |  |
| Any other very stressful event/experience | 32 | 21 | 65.63 | Observing abuse of Geneva Convention | 33 | 6 | 18.18 | |  |  |
| LMHAT |  |  |  | Being responsible for a comrade’s death/injury | 33 | 2 | 6.06 | |  |  |
| Be attacked/ambushed | 34 | 25 | 73.53 | Had a comrade nearby shot/killed | 34 | 13 | 38.24 | |  |  |
| Sighted destroyed homes and villages | 33 | 33 | 100 | Had a close call, dud landed nearby | 34 | 14 | 41.18 | |  |  |
| Receiving small arms fire | 34 | 27 | 79.41 | Had a close call, equipment shot off body | 33 | 0 | 0 | |  |  |
| Seeing dead bodies/human remains | 33 | 25 | 75.76 | Had a close call, was shot/hit but protective gear saved you | 34 | 1 | 2.94 | |  |  |
| Handling human remains | 33 | 17 | 51.52 | Having a member of your own unit become a casualty | 34 | 4 | 11.76 | |  |  |
| Witnessing an accident which resulted in injury/death | 34 | 21 | 61.76 | Being in threatening situations, unable to respond because of RoE | 34 | 15 | 44.12 | |  |  |
| Witnessing violence within the local population | 33 | 27 | 81.81 | Informed others of comrade´s death | 33 | 7 | 21.21 | |  |  |

*Note.* †For the LEC5 the categories ‘happened to me’, ‘witnessed it’, ‘learned about it’, ‘part of my job’ were combined and coded with 1, and the categories ‘not sure’ and ‘doesn’t apply’ were combined and coded as 0. For the LMHAT the 5-point scale asks how often an event was experienced (‘never’, ‘once’, ‘2-4 times’, ‘5-9 times’, ‘more than 10 times’). To keep results comparable with LEC5, we coded never with 0 and combined all other categories coded as 1, so the results display having experienced the respective event at least once. LEC5 = Life Events Checklist for DSM-5; LMHAT = List of the Mental Health Advisory Team; RoE = Rules of engagement.
