# Supplementary material for: Evaluation of an internet-based intervention for service members of the German armed forces with deployment-related posttraumatic stress symptoms
Source: BMC Psychiatry. 2020 May 6;20:205. doi: 10.1186/s12888-020-02595-z (PMC7204035; doi:10.1186/s12888-020-02595-z)
Supplement: Supplementary file 3 — Additional file 3. Supplement 3. Sociodemographic Characteristics for No-shows vs. ITT sample and Dropouts vs. Completers. [file 12888_2020_2595_MOESM3_ESM.docx]

| Supplement 3. Sociodemographic Characteristics for No-shows vs. ITT sample and Dropouts vs. Completers | | | | | | | | | | | | | | | | | |  | |  | |  | |  |  |
| --- | --- | --- | --- | --- | --- | --- | --- | --- | --- | --- | --- | --- | --- | --- | --- | --- | --- | --- | --- | --- | --- | --- | --- | --- | --- |
|  |  |  | | | | Comparison of groups | | | | | |  |  | | |  | | |  | | | | | |  |
| Variables |  | Noshows  (*n* = 6) | Shows (*n* = 31) | | Test statistics  *t (df*) | | *p* | | Dropouts (*n* = 10) | | | | Completers (*n* = 21) | | | Test statistics  *t* (*df*) | | | | | *p* | | | |  |
| Sociodemographic characteristics | | | |  | | | |  | |  |  | |  |  |  | |  | | | | | |  | | |
| Age | *M* (*SD*) | 37.8 (7.2) | 37.7 (10.3) | | -0.04 (9.68) | | . 970 | | 35.5 (8.5) | | | | 38.8 (11.2) | | | 0.90 (23.12) | | | | | .377 | | | |  |
| Education‡ |  |  |  | |  | | 1.000 | |  | | | |  | | |  | | | | | .960 | | | |  |
| Secondary school   qualification | *n* (%) | 1 (16.7) | 5 (16.7) | |  | |  | | 2 (20) | | | | 3 (15) | | |  | | | | |  | | | |  |
| Secondary school   certificate | *n* (%) | 4 (66.7) | 20 (66.7) | |  | |  | | 7 (70) | | | | 13 (65) | | |  | | | | |  | | | |  |
| High school   diploma | *n* (%) | 1 (16.7) | 5 (16.6) | |  | |  | | 1 (10) | | | | 4 (20.0) | | |  | | | | |  | | | |  |
| Joined military | Year (*SD*) | 1998 (8.47) | 2001 (10.2) | | 0.59 (6.20) | | .577 | | 2005 (7.47) | | | | 1999 (11) | | | -1.72 (25.03) | | | | | .097 | | | |  |
| Number of deployments | *M* (*SD*) | 4.67 (5.4) | 2.4 (2.4) | | -1.01 (5.41) | | .356 | | 2.5 (3.95) | | | | 2.35 (1.2) | | | -0.12 (9.88) | | | | | .909 | | | |  |
| Years abroad | *M* (*SD*) | 9 (5.8) | 8.36 (5.2) | | -0.25 (6.8) | | .810 | | 7.62 (3.81) | | | | 8.65(5.7) | | | 0.55 (19.38) | | | | | .587 | | | |  |
| Clinical PTSD (CAPS) |  |  |  | |  | | .368 | |  | | | |  | | |  | | | | | .280 | | | |  |
| No | *n* (%) | 1 (16.7) | 14 (45.2) | |  | |  | | 3 (30) | | | | 11 (52.4) | | |  | | | | |  | | | |  |
| Yes | *n* (%) | 5 (83.3) | 17 (54.8) | |  | |  | | 7 (70) | | | | 10 (47.6) | | |  | | | | |  | | | |  |
| CAPS sum score | *M* (*SD*) | 31 (11.2) | 33.9 (15.7) | | 0.54 (9.27) | | .602 | | 36.4 (15.4) | | | | 32.7 (16) | | | 0.62 (18.47) | | | | | .546 | | | |  |

*Note.* Not all subjects have answered all items, so the number on each item may differ from total. The category “secondary school qualification” (Realschule) also includes “subject-restricted higher education entrance qualification” (Fachhochschulreife). ‡The Fisher′s exact test was used to test the significance of independence in categorical variable. CAPS = Clinician Administered PTSD Scale for DSM-5; df = degrees of freedom; ITT = intention to treat; M = mean; N = sample size; PTSD = posttraumatic stress disorder; SD = standard deviation.
